# The Estimated Prevalence of N-Linked Congenital Disorders of Glycosylation Across Various Populations Based on Allele Frequencies in General Population Databases

**DOI:** 10.3389/fgene.2021.719437

**Published:** 2021-08-10

**Authors:** Sander Pajusalu, Mari-Anne Vals, Laura Mihkla, Ustina Šamarina, Tiina Kahre, Katrin Õunap

**Affiliations:** ^1^Department of Clinical Genetics, Institute of Clinical Medicine, University of Tartu, Tartu, Estonia; ^2^Department of Clinical Genetics, United Laboratories, Tartu University Hospital, Tartu, Estonia; ^3^Children’s Clinic, Tartu University Hospital, Tartu, Estonia

**Keywords:** CDG, congenital disorders of glycosylation, *N*-glycosylation, prevalence, population allele frequencies

## Abstract

Congenital disorders of glycosylation (CDG) are a widely acknowledged group of metabolic diseases. PMM2-CDG is the most frequently diagnosed CDG with a prevalence as high as one in 20,000. In contrast, the prevalence of other CDG types remains unknown. This study aimed to analyze the estimated prevalence of different N-linked protein glycosylation disorders. We extracted allele frequencies for diverse populations from The Genome Aggregation Database (gnomAD), encompassing variant frequency information from 141,456 individuals. To identify pathogenic variants, we used the ClinVar database as a primary source. High confidence loss-of-function variants as defined by the LOFTEE algorithm were also classified as pathogenic. After summing up population frequencies for pathogenic alleles, estimated disease birth prevalence values with confidence intervals were calculated using the Bayesian method. We first validated our approach using two more common recessive disorders (cystic fibrosis and phenylketonuria) by showing that the estimated prevalences calculated from population allele frequencies were in accordance with previously published epidemiological studies. Among assessed 27 autosomal recessive N-glycosylation disorders, the only disease with estimated birth prevalence higher than one in 100,000 was PMM2-CDG (in both, all gnomAD individuals and those with European ancestry). The combined prevalence of 27 different N-glycosylation disorders was around one in 22,000 Europeans but varied considerably across populations. We will show estimated prevalence data from diverse populations and explain the possible pitfalls of this analysis. Still, we are confident that these data will guide CDG research and clinical care to identify CDG across populations.

## Introduction

Congenital disorders of glycosylation (CDG) are a growing group of metabolic diseases with at least 137 defects (Ondruskova et al., 2021). According to the International Classification of Inherited Metabolic Disorders (ICIMD) database, 32 are classified as N-linked protein glycosylation defects (Ferreira et al., 2021). Based on population allele frequencies, the expected birth prevalence of the most common PMM2-CDG could be as high as 1:20,000 (Schollen et al., 2000), and in later reports, 1:77,000 to 1:286,000 (Vals et al., 2018; Yildiz et al., 2020). Mainly at the expense of PMM2-CDG, type 1 defects are more frequently diagnosed than type 2 defects (Peanne et al., 2017; Medrano et al., 2019) but compared to PMM2-CDG, other defects occur much seldom. The estimated combined prevalence of CDG among the Saudi population is 14 per million (Alsubhi et al., 2017), whereas, in Poland, the prevalence is approximately one case per million (Lipinski et al., 2021). The worldwide individual prevalence of different defects remains unknown.

The emergence of large-scale population-based exome and genome sequencing studies and the aggregation into even more extensive cross-population databases like gnomAD makes allele frequency data available from more than 100,000 individuals across different populations (Karczewski et al., 2020). Disease prevalence can be estimated when these data are combined with clinical variant classification databases like ClinVar (Landrum et al., 2018) and variant loss-of-function predictions. This approach has been previously used on assessing disease prevalence of different limb-girdle muscular dystrophy subtypes across populations (Liu et al., 2019). Although direct calculations using the Hardy-Weinberg equation can be used, the methods developed by Liu et al. (2019) make use of more advanced Bayesian statistics to add confidence intervals on prevalence estimators.

This study aimed to analyze the estimated prevalence of N-linked protein glycosylation defects across different populations based on allele frequencies in general population databases.

## Materials and Methods

Only N-linked protein glycosylation defects with autosomal recessive inheritance patterns (27/32) were included in the analysis. The list was based on the ICIMD database (Ferreira et al., 2021). We extracted allele frequencies for different populations from The Genome Aggregation Database (gnomAD v2.1.1) (Karczewski et al., 2020), encompassing variant frequency information from 141,456 individuals. We defined variants as pathogenic if they were classified pathogenic or likely pathogenic in the ClinVar database (version 20210119) (Landrum et al., 2018) or were marked as high confidence loss-of-function (Moller et al., 2016) variants by the LOFTEE algorithm (Karczewski et al., 2020). In the case of conflicting annotations, high confidence LoF variants marked as (likely) benign in ClinVar were considered as non-pathogenic. Also, variants not passing all quality filters in the gnomAD variant call set were eliminated from counting toward the sum of pathogenic alleles.

The calculations were carried out across all seven gnomAD populations (African/African American, Latino/Admixed American, non-Finnish European, Finnish, Ashkenazi Jewish, East Asian, and South Asian). The number of individuals per population ranged from 5,185 (Ashkenazi Jewish) to 64,603 (non-Finnish European). In addition, we calculated prevalence estimates for the Estonian population (2,418 individuals), which is a subpopulation of non-Finnish Europeans. After obtaining population frequencies for pathogenic alleles, estimated disease birth prevalence values with confidence intervals were calculated using Bayesian statistics adapted from the previously published study by Liu et al. (2019). No manual variant curation was performed, and no gene-based exceptions were made to assure reproducibility and robustness (i.e., for PMM2-CDG, the p.Arg141His variant homozygotes were not excluded although known to be embryonically lethal). For validation, we used two common recessive disorders (cystic fibrosis and phenylketonuria) and compared the estimated disease prevalence with the data from epidemiological studies conducted in different populations.

## Results

The estimated prevalence from population allele frequencies of cystic fibrosis and phenylketonuria was in accordance with previously published data (Supplementary Table 1). Results from published epidemiological studies fell into 95% confidence intervals in non-Finnish European, Estonian, and Finnish populations, where reliable population prevalence is known.

The selected list of N-linked protein glycosylation defects, their estimated prevalence, and the number of reported cases is presented in Table 1. The full list of all defects in all assessed populations is shown in Supplementary Table 2.

**TABLE 1:** N-linked protein glycosylation defects with highest estimated prevalence.

Defect	Population	Estimated prevalence	Reported patients (references)
PMM2-CDG	NFE FIN EST AFR AMR EAS SAS ASJ	1:27,465 (1:24,014-1:31,804) 1:18,745 (1:14,391-1:25,744) 1:61,506 (1:30,827-1:213,327) 1:288,214 (1:177,276-1:582,250) 1:64,498 (1:47,771-1:93,421) 1:150,016 (1:94,205-1:290,357) 1:366,999 (1:229,546-1:716,111) 1:19,908 (1:13,368-1:33,911)	>900 (Altassan et al., 2019)
FUK-CDG	NFE FIN EST AFR AMR EAS SAS ASJ	1:491,021 (1:376,701-1:675,057) 1:20,817,888 (1:7,099,020-1:997,371,113) 1:1,163,396 (1:371,284-1:113,077,583) 1:140,528 (1:92,099-1:250,277) 1:159,571 (1:110,439-1:257,822) 1:12,248 (1:9,355-1:16,948) 1:264,096 (1:170,511-1:483,909) 1:255,447 (1:129,730-1:848,576)	2 (Ng et al., 2018)
ALG6-CDG	NFE FIN EST AFR AMR EAS SAS ASJ	1:623,512 (1:471,533-1:875,344) 1:31,451,234 (1:10,034,414-1:3,065,715,982) 1:3,891,489 (1:1,025,904-1:1,609,067,019) 1:1,819,171 (1:898,773-1:6,624,417) 1:13,909,852 (1:5,657,192-1:128,018,704) 1:66,077,105 (1:17,423,193-1:27,292,863,020) 1:7,088,553 (1:3,056,117-1:46,137,181) 1:17,848,406 (1:4,706,371-1:7,371,298,516)	101 (Peanne et al., 2017)
MAN2B2-CDG	NFE FIN EST AFR AMR EAS SAS ASJ	1:787,319 (1:586,411-1:1,130,745) 1:52,368,616 (1:15,379,043-1:10,698,108,148) 1:1,945,432 (1:571,159-1:398,260,061) 1:399,033 (1:237,158-1:868,735) 1:995,113 (1:577,663-1:2,287,515) 1:11,323 (1:8,713-1:15,500) 1:104,832 (1:73,327-1:166,377) -	1 (Verheijen et al., 2020)
ALG1-CDG	NFE FIN EST AFR AMR EAS SAS ASJ	1:881,984 (1:651,920-1:1,281,505) 1:4,775,806 (1:2,058,960-1:31,088,465) 1:3,882,716 (1:1,023,873-1:1,603,070,711) 1:329,069 (1:199,633-1:684,648) 1:2,981,452 (1:1,514,011-1:9,907,069) 1:2,543,998 (1:1,124,720-1:14,579,668) 1:1,559,334 (1:828,918-1:4,508,711) 1:47,656 (1:29,448-1:95,348)	57 (Ng et al., 2016)
MPI-CDG	NFE FIN EST AFR AMR EAS SAS ASJ	1:1,294,251 (1:930,944-1:1,962,331) 1:4,778,023 (1:2,059,807-1:31,111,585) 1:11,654,073 (1:2,660,508-1:8,720,690,758) 1:8,630,916 (1:3,389,920-1:102,061,250) 1:22,366,551 (1:8,439,237-1:371,016,597) 1:13,209,464 (1:4,505,222-1:631,735,293) 1:12,962,840 (1:5,091,807-1:153,179,670) 1:53,570,813 (1:12,229,235-1:40,091,239,613)	35 (Cechova et al., 2020)
ALG8-CDG	NFE FIN EST AFR AMR EAS SAS ASJ	1:1,415,559 (1:1,011,381-1:2,169,560) 1:113,720 (1:76,487-1:193,148) 1:1,167,178 (1:372,380-1:113,784,408) 1:467,544 (1:273,118-1:1,059,029) 1:2,983,716 (1:1,515,013-1:9,917,690) 1:5,516,877 (1:2,166,449-1:65,327,618) 1:8,491,922 (1:3,562,236-1:64,394,419) 1:53,570,813 (1:12,229,235-1:40,091,239,613)	19 (Hock et al., 2015; Bastaki et al., 2018; Vuillaumier-Barrot et al., 2019)
ALG12-CDG	NFE FIN EST AFR AMR EAS SAS ASJ	1:3,351,728 (1:2,231,972-1:5,796,068) 1:7,006,414 (1:2,848,761-1:64,600,402) 1:11,653,895 (1:2,660,472-1:8,720,505,722) 1:11,091,299 (1:4,185,867-1:183,600,265) 1:1,785,729 (1:967,871-1:4,889,276) 1:19,815,990 (1:6,323,450-1:1,927,830,733) 1:46,648,320 (1:14,885,720-1:4,538,726,166) 1:17,888,161 (1:4,715,426-1:7,399,706,924)	15 (Sturiale et al., 2019; Tahata et al., 2019; De la Morena Barrio et al., 2020; Lekka et al., 2021)
RFT1-CDG	NFE FIN EST AFR AMR EAS SAS ASJ	1:4,002,089 (1:2,622,918-1:7,127,436) 1:104,751,692 (1:27,620,721-1:43,268,721,479) 1:3,882,747 (1:1,023,880-1:1,603,094,646) 1:6,903,683 (1:2,808,018-1:63,498,158) 1:694,726 (1:419,403-1:1,460,905) 1:19,816,478 (1:6,323,581-1:1,927,952,708) 1:5,130,463 (1:2,320,228-1:26,453,803) 1:53,571,026 (1:12,229,292-1:40,091,317,606)	17 (Abiramalatha et al., 2019; Quelhas et al., 2019)
MAN1B1-CDG	NFE FIN EST AFR AMR EAS SAS ASJ	1:6,787,005 (1:4,228,673-1:13,348,564) 1:104,751,828 (1:27,620,743-1:43,268,894,067) 1:1,940,866 (1:570,034-1:396,154,895) 1:1,347,137 (1:692,613-1:4,304,308) 1:4,603,156 (1:2,204,053-1:18,769,689) 1:13,235,145 (1:4,512,603-1:635,115,108) 1:31,096,221 (1:10,605,563-1:1,487,347,436) -	40 (Balasubramanian et al., 2019)
MOGS-CDG	NFE FIN EST AFR AMR EAS SAS ASJ	1:9,149,993 (1:5,524,081-1:19,239,011) 1:31,246,156 (1:9,968,990-1:3,045,708,636) - 1:5,305,643 (1:2,225,824-1:40,213,116) 1:29,725,472 (1:10,706,387-1:781,475,013) 1:6,817,673 (1:2,572,492-1:113,059,182) 1:3,889,994 (1:1,830,265-1:16,972,762) -	12 (Anzai et al., 2021; Lo Barco et al., 2021)

*Population abbreviations as defined in gnomAD: NFE, non-Finnish European; FIN, Finnish; EST, Estonian; AFR, African/African American; AMR, Latino/Admixed American; EAS, East Asian; SAS, South Asian; ASJ, Ashkenazi Jewish.*

PMM2-CDG showed the highest estimated prevalence counted from 71 different pathogenic variants, and it is more prevalent in European (1 in 27,000), Ashkenazi Jewish (1 in 20,000), and admixed American populations (1 in 64,000). All other N-linked protein glycosylation defects had a much lower prevalence.

FUK-CDG and MAN2B2-CDG, which are both only recently reported and classified as N-linked protein glycosylation defects, showed high estimated prevalence, especially in East-Asians where FUK-CDG prevalence was estimated at 1:12,000 and MAN2B2-CDG at 1:11,000.

The estimated prevalence of combined N-linked protein glycosylation defects in Europeans was one in 22,000 or one in 24,000 after excluding FUK-CDG and MAN2B2-CDG. In East-Asians, however, the total CDG prevalence was estimated at 1:5,613, dropping to 1:121,935 after excluding FUK-CDG and MAN2B2-CDG. CDG seems to be more common in Finnish (1:16,000) and Ashkenazi Jewish populations (1:14,000). In Estonians, the combined CDG birth prevalence is estimated at around 1:50,000.

## Discussion

This study presents the estimated prevalence of different N-linked protein glycosylation defects calculated from population allele frequencies. As the CDG group involves at least 137 defects, we emphasize that we only included 27 autosomal recessive protein N-glycosylation affecting defects and excluded defects that affect multiple glycosylation pathways.

The used methods make several assumptions and do not take variant- and gene-specific properties into account. For example, the calculations assume that pathogenic variants are independent and not on the same allele. Also, Hardy-Weinberg equilibrium is assumed but may not be present in some populations with more consanguinity. All diseases are considered to result from the biallelic loss-of-function mechanism; however, we cannot exclude the possibility that for some CDG, only specific variants (e.g., certain missense variants) cause the disease.

Also, this method did not consider the possibility that some variant recombinants might not be compatible with life. Since we cannot exclude this phenomenon in these genes, the chance of p.Arg141His homozygosity was also included when calculating the estimated prevalence of PMM2-CDG. However, as seen from the subanalysis of the Estonian population, it does not change the prevalence that much. In Estonia, the estimated prevalence of PMM2-CDG with the exclusion of p.Arg141His homozygosity was 1:77,000, and with inclusion, 1:62,000 (Vals et al., 2018). This example shows that the estimated prevalence of different defects might be somewhat overrated.

On the other hand, some aspects can cause an underestimation of disease prevalence. For example, currently, the data relies on gnomAD variants, and thus ultra-rare pathogenic variants not seen in gnomAD are not counted toward prevalence estimation. Also, only small variants (single nucleotide substitutions and small indels) were included, and thus pathogenic copy-number variants (deletions) may raise the disease prevalence estimators. Regarding missense variants, only those classified as pathogenic in ClinVar were included, while some other missense, as well as synonymous, intronic, and regulatory variants, may truly be pathogenic but missed due to lack of such information in databases. Regarding missense variants, one possibility to improve the estimates would be to include variants with high pathogenicity predictions. However, as shown by Liu et al. (2019) and consistent with our experience (data not shown), this leads to an overestimation of prevalence. Thus, we did not include any missense variant prediction tools in our final calculations. To be on the conservative side, we eliminated all low-quality variants in gnomAD; however, some of them, especially indels, may be true and pathogenic. Of note, presented results rely on data extracted from two publicly available databases, ClinVar and gnomAD, at a single time point. If other databases would have been used, we expect to see slightly different results; however, confidence intervals are shown to address this variability. Moreover, as the databases update both allele frequencies and pathogenicity classifications, a follow-up study in the future is planned to investigate whether the prevalence estimates will change after some years.

Despite many pitfalls, the validation with two well-known recessive diseases—cystic fibrosis and phenylketonuria—proved the method’s accuracy and usability for estimating the disease prevalence. From our previous epidemiological studies in Estonia, the prevalence of phenylketonuria is known to be 1:6,700 (Lillevali et al., 2018) correlating with the estimate from gnomAD data is 1:6,604 (95% CI 1:4,269-1:12,073). For cystic fibrosis, the birth prevalence in Estonia is 1:7,743 (Kahre, 2004) comparing well with the calculated estimate 1:8,482 (95% CI 1:5,351-1:16,269). For other well-studied populations like Europeans and Finnish, the data is in good correlation as well (Supplementary Table 1).

As shown in Table 1, compared to PMM2-CDG, all other N-linked protein glycosylation defects are less common. Still, our reported prevalence of different defects shows compliance with published clinical observations. In 2016, an informal inquiry was conducted in various European laboratories to evaluate the number of screening positive and molecularly confirmed CDG-I and CDG-II patients (Peanne et al., 2017). Based on their results, the most common type 1 N-glycosylation defect in Europe was PMM2-CDG, followed by ALG6-CDG, ALG1-CDG, and MPI-CDG. If we compare this distribution with non-Finnish Europeans, a similar pattern is seen with type 1 N-glycosylation defects. The most common type 2 N-glycosylation defects, according to the inquiry, were MAN1B1-CDG, MGAT2-CDG, and B4GALT1-CDG. In the non-Finnish European population, MAN1B1-CDG is followed by MOGS-CDG, which does not show an abnormal serum transferrin profile with routine CDG screening (De Praeter et al., 2000), and therefore was not reported by laboratories. Compared to these two defects, MGAT2-CDG and B4GALT1-CDG show a much lower prevalence.

Unexpectedly, two recently described defects, FUK-CDG and MAN2B2-CDG, showed the prevalence of higher than one per million. To date, FUK-CDG is described only in two individuals, whereas MAN2B2-CDG only in one (Ng et al., 2018; Verheijen et al., 2020). For *FUK*, we found 119, and for *MAN2B2*, 84 different possibly disease-causing alleles matching our definition of pathogenicity. Disagreement between relatively high estimated prevalence and the low number of reported cases could be explained by the phenomenon where homozygosity or compound heterozygosity of some variants may not be viable, as is the case with p.Arg141His homozygosity in *PMM2* (Matthijs et al., 1998). Also, *FUK* and *MAN2B2* are not included in smaller panels and are only identified by whole-exome sequencing. Theoretically, it may be the cause for the underdiagnosis of these defects to some extent. Notably, one of the pathogenic missense variants reported by Ng et al. (2018), NM_145059.3:c.2980A > C (p.Lys994Gln), reaches allele frequency of 0.002 in the East Asian population, and there is one homozygous individual present in gnomAD. Although mild phenotypes cannot be excluded in gnomAD individuals, also non-pathogenicity of this allele, at least in the homozygous state, should be considered. Of note, *FUK* and *MAN2B2* both lack homozygous LoF variant carriers in gnomAD, which hints that biallelic LoF may be an actual pathogenic mechanism for those genes.

Unlike FUK-CDG and MAN2B2-CDG, other recently described defects (GFUS-CDG, OSTC-CDG, and FUT8-CDG) show lower prevalence.

Although the individual prevalence of each N-linked protein glycosylation defect is very low, the combined prevalence for this group of CDG is notable. If all 27 defects are included, the combined prevalence in non-Finnish Europeans is one in 22,000. If we exclude FUK-CDG and MAN2B2-CDG, the prevalence in Europeans is slightly lower, one in 24,000. Compared to available data (Vals et al., 2018; Lipinski et al., 2021), the expected and observed prevalence differ. The lower observed prevalence might be somewhat underestimated because of undiagnosed patients due to different causes such as unrecognized phenotypes, negative screening results or limited access to different metabolic and molecular studies. In the Estonian population, the combined prevalence of 25 defects is 1:50,000. Therefore, with a yearly birth rate of 13,000–14,000, one individual with protein N-glycosylation defect should be born every 4 years correlating with our clinical data.

In summary, we broaden the knowledge about N-linked protein glycosylation disorders, which hopefully helps raise awareness about the prevalence of this CDG subgroup.

## Data Availability Statement

Publicly available datasets were analyzed in this study. These data can be found at: https://gnomad.broadinstitute.org/ and https://www.ncbi.nlm.nih.gov/clinvar/.

## Author Contributions

SP planned and conducted the study, carried out the statistical analysis, and compiled the manuscript. M-AV planned, conducted the study, analyzed the data, and compiled the manuscript. LM conducted literature review, analyzed the data, and reviewed the manuscript. UŠ and TK analyzed the data and reviewed the manuscript. KÕ planned and conducted the study and reviewed the manuscript. All authors contributed to the article and approved the submitted version.

## Conflict of Interest

The authors declare that the research was conducted in the absence of any commercial or financial relationships that could be construed as a potential conflict of interest.

## Publisher’s Note

All claims expressed in this article are solely those of the authors and do not necessarily represent those of their affiliated organizations, or those of the publisher, the editors and the reviewers. Any product that may be evaluated in this article, or claim that may be made by its manufacturer, is not guaranteed or endorsed by the publisher.
