# Supplementary material for: The Estimated Prevalence of N-Linked Congenital Disorders of Glycosylation Across Various Populations Based on Allele Frequencies in General Population Databases
Source: Front Genet. 2021 Aug 10;12:719437. doi: 10.3389/fgene.2021.719437 (PMC8383291; doi:10.3389/fgene.2021.719437)
Supplement: Supplementary file 1 [file Table_1.DOCX]

Supplementary Material

# Supplementary Table 1. The prevalence of phenylketonuria and cystic fibrosis across various populations from published epidemiological studies compared to the prevalence estimators calculated from gnomAD database allele frequencies.

# The prevalence of phenylketonuria. Population abbreviations as defined in gnomAD: NFE – non-Finnish European, FIN – Finnish, EST – Estonian, AFR – African/African American, AMR – Latino/Admixed American, EAS – East Asian, SAS – South Asian, ASJ – Ashkenazi Jewish

| **Population** | **Reported prevalence from epidemiological studies** | **Ref** | **Estimated prevalence (gnomAD)** | **95% confidence interval** |
| --- | --- | --- | --- | --- |
| **NFE** | 1:10,000  1:10,000-1:15,000  1:2,622-1:4,5871  1:8,034  1:8,771 | [1]  [2]  [3]  [4]  [5] | 1:6,569 | 1:5,973-1:7,270 |
| **FIN** | 1:200,000 | [2] | 1:146,959 | 1:96,582- 1:260,373 |
| **EST** | 1:6,010  1:6,700 | [6]  [7] | 1:6,604 | 1:4,269-1:12,073 |
| **AFR** | 1:90,909 (Black/Black British)  1:100,000 (African Americans) | [5]  [8] | 1:93,667 | 1:63,957-1:154,904 |
| **AMR** | 1:9,615-1:20,833 (Pan America) | [3] | 1:21,874 | 1:17,302-1:28,802 |
| **EAS** | 1:17,000 (China)  1:125,000 (Japan)  1:15,923 (China) | [2]  [2]  [9] | 1:29,295 | 1:21,154-1:44,141 |
| **SAS** | 1:34,482 (Asian/Asian British) | [5] | 1:26,639 | 1:20,492-1:36,481 |
| **ASJ** | 1:12,500 (Jews)  1:5,000 (Yemenite Jews)  1:5,300 (Yemenite Jews) | [10]  [10]  [2] | 1:1,101 | 1:895-1:1,397 |

# The prevalence of cystic fibrosis. Population abbreviations as defined in gnomAD: NFE – non-Finnish European, FIN – Finnish, NFE-EST – Estonian, AFR – African/African American, AMR – Latino/Admixed American, EAS – East Asian, SAS – South Asian, ASJ – Ashkenazi Jewish

| **Population** | **Reported prevalence from epidemiological studies** | **Ref** | **Estimated prevalence (gnomAD)** | **95% confidence interval** |
| --- | --- | --- | --- | --- |
| **NFE** | 1:2,000-3,000  1:4,500-1:6,000 | [11]  [12] | 1:2,463 | 1:2,286-1:2,664 |
| **FIN** | 1:25,000  1:30,000 | [11] [12]  [13] | 1:27,495 | 1:20,592-1:39,163 |
| **EST** | 1:7,743 | [14] | 1:8,482 | 1:5,351-1:16,269 |
| **AFR** | 1:7,056  1:15,100 (African Americans) | [11]  [15] | 1:13,668 | 1:10,675-1:18,318 |
| **AMR** | 1:8,500 (Mexico)  1:3,900 (Cuba)  1:8,000-1:10,000 (Latin and South America)  1:6,100 (Argentina)  1:15,000 (Costa Rica) | [11]  [11]  [12]  [12]  [12] | 1:11,916 | 1:9,723-1:15,048 |
| **EAS** | 1:100,000-1:350,000 (Japan) | [11] | 1:129,090 | 1:82,251-1:242,706 |
| **SAS** | 1: 10,000 (Asians living in the UK - mainly Indian/Pakistani)  1: 40,000 (Asians living in the USA)  1:40,000-100,000 (India) | [11]  [11]  [11] | 1:17,472 | 1:13,770-1:23,122 |
| **ASJ** | 1:5,000 (Israel) | [16] | 1:3,125 | 1:2,402-1:4,283 |

**References**

1. van Wegberg, A.M.J., et al., *The complete European guidelines on phenylketonuria: diagnosis and treatment.* Orphanet Journal of Rare Diseases, 2017. **12**(1).

2. Williams, R., C.D. Mamotte, and J.R. Burnett, *Phenylketonuria: An Inborn Error of Phenylalanine Metabolism.* Clin Biochem Rev, 2008. **29**: p. 31-41.

3. Shoraka, H.R., et al., *Global prevalence of classic phenylketonuria based on Neonatal Screening Program Data: systematic review and meta-analysis.* Clin Exp Pediatr, 2020. **63**(2): p. 34-43.

4. Loeber, J.G., *Neonatal screening in Europe; the situation in 2004.* J Inherit Metab Dis, 2007. **30**(4): p. 430-8.

5. Hardelid, P., et al., *The birth prevalence of PKU in populations of European, South Asian and sub-Saharan African ancestry living in South East England.* Ann Hum Genet, 2008. **72**(Pt 1): p. 65-71.

6. Õunap, K., et al., *Development of the phenylketonuria screening programme in Estonia.* J Med Screen, 1998. **5**: p. 22-23.

7. Lillevali, H., et al., *Hyperphenylalaninaemias in Estonia: Genotype-Phenotype Correlation and Comparative Overview of the Patient Cohort Before and After Nation-Wide Neonatal Screening.* JIMD Rep, 2018. **40**: p. 39-45.

8. Gjetting, T., et al., *A phenylalanine hydroxylase amino acid polymorphism with implications for molecular diagnostics.* Mol Genet Metab, 2001. **73**(3): p. 280-4.

9. Xiang, L., et al., *Phenylketonuria incidence in China between 2013 and 2017 based on data from the Chinese newborn screening information system: a descriptive study.* BMJ Open, 2019. **9**(8): p. e031474.

10. Bercovich, D., et al., *A mutation analysis of the phenylalanine hydroxylase (PAH) gene in the Israeli population.* Ann Hum Genet, 2008. **72**(Pt 3): p. 305-9.

11. WHO, *The molecular genetic epidemiology of cystic fibrosis. Report of a joint meeting of WHO/ECFTN/ICF(M)A/ECFS*. 2004.

12. Scotet, V., C. L'Hostis, and C. Ferec, *The Changing Epidemiology of Cystic Fibrosis: Incidence, Survival and Impact of the CFTR Gene Discovery.* Genes (Basel), 2020. **11**(6).

13. Aula, P.K., H.; Palotie, A.; eritoimetaja Mikelsaar, A.-V., *Pärilikkusmeditsiin*, ed. A.-V. eritoimetaja Mikelsaar. 2010: Medicina.

14. Kahre, T., *Cystic Fibrosis in Estonia*, in *Department of Biology, Institute of Molecular and Cell Biology*. 2004, University of Tartu: Tartu.

15. Owusu, S.K., et al., *Cystic fibrosis in black African children in South Africa: a case control study.* Journal of Cystic Fibrosis, 2020. **19**(4): p. 540-545.

16. Shoshani, T., *Association of a Nonsense Mutation (WI 282X), the Most Common Mutation in the Ashkenazi Jewish Cystic Fibrosis Patients in Israel, with Presentation of Severe Disease.* Am. J. Hum. Genet, 1992. **50**: p. 222-228,.
